# Assessment of health-related quality of life of patients with gout and hyperuricemia in the Tianjin area according to the SF-6Dv2 scale

**DOI:** 10.3389/fpubh.2026.1744513

**Published:** 2026-02-24

**Authors:** Xiaohui Zheng, Baoyu Wang, Zhuang Cui, Shi-Tong Xie, Zhenying Zhao

**Affiliations:** 1 Department of Pharmacy, Tianjin Union Medical Center, The First Affiliated Hospital of Nankai University, Tianjin, China; 2 Endocrinology Department, Tianjin Union Medical Center, The First Affiliated Hospital of Nankai University, Tianjin, China; 3 School of Public Health, Tianjin Medical University, Tianjin, China; 4 School of Pharmaceutical Science and Technology, Tianjin University, Tianjin, China

**Keywords:** cross-sectional study, gout and hyperuricemia, SF-6Dv2 scale, Tianjin area, utility value

## Abstract

**Background:**

The number of patients with hyperuricemia and gout is constantly rising and the conditions are seriously affecting their quality of life. Thus, it is of great importance to identify a assessment scale to assess the quality of life suitable for the Chinese population. The aim of this study was to evaluate the health-related quality of life (HRQoL) of patients with hyperuricemia and gout in Tianjin, using the Short Form of 6-Dimensions Health 2nd version (SF-6Dv2) scale, and to explore its influencing factors.

**Methods:**

A total of 171 patients who visited the hyperuricemia gout clinic in a Class III and Grade A hospital were collected from September 2022 to May 2024. The SF-6Dv2 scale was completed by face-to-face interview and its utility value was measured. Sex- and age-matched individuals from the community and a healthy population on physical examination from the same hospital served as the control group. This study compared the HRQoL of respondents through the SF-6Dv2 score and explored its influencing factors.

**Results:**

A total of 171 patients (mean [SD] age: 45.34 [16.60] years) and 171 respondents (mean [SD] age: 41.24[16.60] years) from the control population were included. The mean utility value of the disease group was 0.671 (0.270), which was significantly lower than that of the control group (0.803 [0.155], *p* < 0.001). In terms of physical function, role limitation, social function, pain, mental health, and vitality, patients reported worse quality of life than the control group. The results of multiple linear regression analysis showed that patients with normal triglyceride levels, who disliked seafood, had single marital status, and mild hyperuricemia with gout had higher health utility value.

**Conclusion:**

This study found that the utility value of the SF-6Dv2 scale for patients with hyperuricemia was lower than that of the control group. Furthermore, the level of triglycerides, the preference for seafood, the severity of hyperuricemia and gout, and marital status of the patients were correlated with the HRQoL of the patients with hyperuricemia and gout. This findings of this study have important practical value.

## 1 Introduction

Hyperuricemia is a metabolic abnormality syndrome caused by purine metabolism disorder. When the level of uric acid in the human body blood exceeds its saturation in the blood or tissue fluid, sodium urate crystals form locally in joints and deposit therein, inducing local inflammatory responses and destroying tissues, thus developing to gout. When the level of patient’s uric acid concentration remains high for a long time, urate salts deposit in the kidneys, joints, and other regions, which can lead to arthritis, chronic kidney disease, and other conditions, causing great pain to the patient (1, 2). Today, with the improvement of living standards, the incidence and prevalence of hyperuricemia and gout is continuously increasing worldwide (3). According to the literature (4), in Jiangsu, Zhejiang, and Shanghai, the economically developed coastal areas of China, the prevalence of patients with hyperuricemia and gout is significantly higher than in other inland areas. Studies have shown (5) that the number of patients with hyperuricemia and gout in China is constantly increasing and has become the most common metabolic disease following diabetes. Hyperuricemia and gout can cause cardiovascular disease and, in severe cases, can impact on quality of life of individuals and impose a heavy burden of disease (6, 7).

In recent years, health-related quality of life (HRQoL) has received increasing attention. Quality of life scales are used a measurement tool for health outcomes and can evaluate the prevention and treatment effects of chronic diseases in patients. Quality of life assessment is applied mainly in the economic evaluation of clinical trials and medical intervention measures, providing an important basis for the formulation of health policies and the efficient allocation of health resources (8–10). Therefore, the application of HRQoL scales is of significant clinical significance. The quality of life scale encompasses multi-dimensional evaluations of an individual’s physical health, mental health, social function, spirit, and personal role, reflecting the comprehensive impact of diseases on the quality of health status of an individual (11, 12). Quality of life scales are widely used in Europe and America, but its application in China have been more tardive in comparison. In China, the quality of life of residents with hematological diseases, diabetes, psoriasis, hypertension, myopia, fibrodysplasia ossificans progressiva, immune thrombocytopenia, and have been reported (13–19). Currently, HRQoL scales have been developed for investigations examining gout, and studies on the quality of life of patients, such as the GAQ scale and TIQ-20. However, none of these scales is specifically designed for Chinese population (20, 21). Furthermore, life scales related to hyperuricemia and relevant research reports are currently unavailable.

## 2 Patients and methods

### 2.1 Patients

This study was conducted in the Tianjin area. The patient population focused on outpatients with hyperuricemia and gout visiting a large tertiary hospital in Tianjin from September 2022 to May 2024. The control group consisted of community residents who had been working and living in Tianjin for a prolonged period and individuals who underwent health checks at the same hospital. The studies involving human participants were reviewed and approved by the Ethics Committee of the Tianjin Union Medical Center (Fast Review No. B137 of 2024). The participants provided their written informed consent to participate in this study. The inclusion of the patient group and the general population in this study met the following criteria: (i) age ≥18 years; (ii) residents who had been working or living in Tianjin for a long time; (iii) no cognitive dysfunction and could complete the questionnaire by themselves; and (iv) participated freely in the study. The following exclusion criteria were applied: (i) age <18 years; (ii) residents seeking medical treatment in a different location or those who could not live in Tianjin for a prolonged period time; (iii) Those with cognitive dysfunction or mental illness are not allowed to read the questionnaire by themselves.

### 2.2 Investigation model

This study adopted cross-sectional research model and the researchers and respondents were investigated “face-to-face.” A total of 171 patients with hyperuricemia and gout were included in the study. The information collected from the included population consisted of demographic characteristics and relevant clinical information (sex, age, ethnicity, marital status, educational background, and occupation) Monthly income, medical insurance status, smoking history, drinking history, family history of chronic diseases, staying up late, personal disease history, BMI index, triglyceride level, preference for seafood, duration of hyperuricemia and gout, severity of hyperuricemia and gout. The sampling time for the control group was the same as that for the disease group, and the number of people in both groups was the same, with no differences in age or sex. Both study groups completed the SF-6Dv2 scale through face-to-face interviews. The patient group and the control group were matched according to age and sex to evaluate the applicability of the Chinese version of the SF-6Dv2 scale in assessing the quality of life of gout patients with hyperuricemia in Tianjin and to explore related influencing factors associated with quality of life.

### 2.3 Methods

This study used the simplified version of the SF-6Dv2 scale developed for the Chinese population. Compared with other HRQoL scales, respondents spent less time completing this scale. This scale has been verified previously. The respondents indicated that there was no comprehension barrier during the process of completing the questionnaire. The questionnaire was easy for Chinese people to read and had no floor/ceiling effect (22, 23). In addition, the scale has been widely applied in some studies, such as in assessing the quality of life of patients with blood disorders and in the general population (15, 16). Thus, this study used this scale to assess the quality of life of patients with hyperuricemia and gout to investigate the applicability of the scale. Furthermore, since this investigation was based on the measurement of general health preferences of research subjects, it was able to generate health utility values to calculate pharmacoeconomics, and Quality-adjusted life years (QALYs) (24). Based on the literature (25), the method for calculating the health utility value in this study was the TTO method. Face-to-face interviews were conducted among the general population of multiple cities in China to collect basic demographic characteristics. The responses of the respondents were then modeled to obtain the coefficients at each dimension level. Meanwhile, researchers controlled the quality of the collected data. Complete health status was set at 1. The utility value of the health status was obtained by subtracting the coefficients of each dimension level calculated from 1, thereby allowing to estimate the utility value of the health status. The corresponding horizontal coefficients for each dimension can be obtained in the literature (25). The utility value in pharmacoeconomics usually ranges from 0 to 1, and sometimes it may be negative, representing a worse health condition than death. It can reflect the patient’s subjective satisfaction with their own health status, thereby helping doctors assess the patient’s health status better, make clinical decisions, and at the same time, it is conducive to optimizing the allocation of health resources.

The Chinese version of the SF-6Dv2 scale was used to compare patients with gout with hyperuricemia and healthy individuals. The SF-6Dv2 scale was developed based on the SF-6D scale. The SF-6Dv2 scale is divided into six dimensions, namely physical function, role limitation, social function, pain, mental health, and vitality. Each dimension is divided into 5 to 6 grades, with higher grades indicating more severe diseases. A total of 18,750 health status were described. The SF-6Dv2 marked the disease level of each dimension with numbers. The numbers of these six dimensions form a six-digit code representing a person’s health status. Each dimension was then divided into 5 to 6 grades, with higher grades indicating more severe diseases. A total of 18,750 health states were described. SF-6Dv2 marks the disease level of each dimension with numbers. The range of health utility values of the Chinese version of SF-6Dv2 was from −0.277 (the worst health status, having the digital code “555655”) to 1 (the best health status, with the digital code “111111”) (25).

### 2.4 Statistical analysis

All statistical analyses in this study were conducted using SPSS v.23.0 software. Non-parametric Mann–Whitney *U* Tests were used to analyze continuous variables, and chi-square tests were used to evaluate differences in the proportions of categorical variables. The educational level, sex, age, ethnicity, marital status, occupation, monthly income, medical insurance status, smoking history, drinking history and family history of chronic diseases of the two groups was evaluated using Fisher’s exact test. In this study, utility values were calculated using the integral utility system as described previously (25). The descriptive statistics were described by the proportion (%), average value, and standard deviation. All the *p*-values reported in this study were two-tailed and statistically significance was considered at *p*-values of <0.05.

## 3 Results

### 3.1 Data collection and characteristics

#### 3.1.1 Collection of sample data

**Figure 1:**
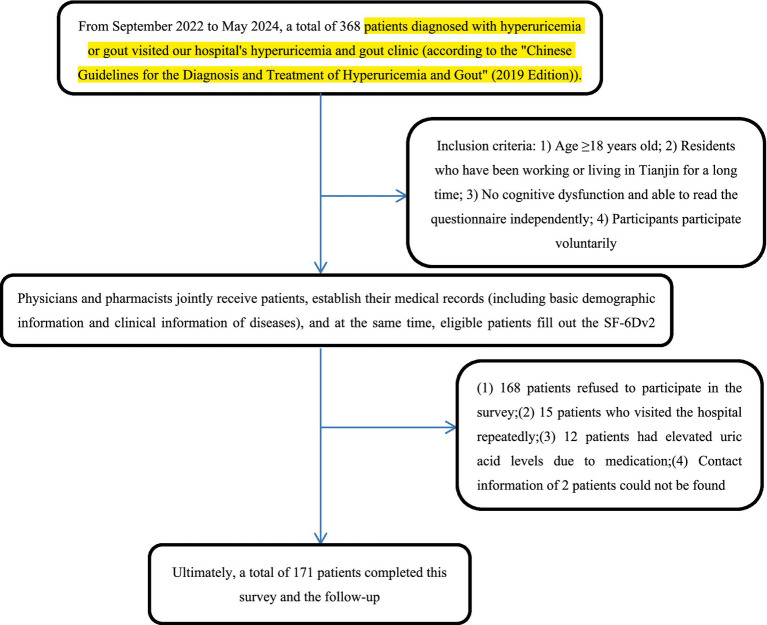
The flowchart of the patients included.

From September 2022 to May 2024, the hyperuricemia gout outpatient department of the participating tertiary hospital received a total of 368 confirmed patients with gout. According to the inclusion criteria of this study, pharmacists registered the medical records for each patient. During the survey, 168 patients refused to participate. Among them, 15 patients repeatedly returned for follow-up visits due to hyperuricemia and gout. Twelve patients reported a transient increase in uric acid caused by taking medication. Two patients did not register their contact information and therefore could not be followed up later. Ultimately, a total of 171 patients with hyperuricemia and gout were included and followed. Figure 1 shows the flow chart of the included patients.

#### 3.1.2 Characteristics of the sample personnel

The basic demographic information of the included patients is shown in Table 1. The average age was 45.34 (16.60) years and the proportion of male patients was 90.06%, which is consistent with the literature (3). The incidence of male patients with hyperuricemia and gout is more common. The Han population comprised the majority, accounting for 93.57%. Married patients accounted for 77.19%, and more than half of them had received higher education (57.89%). The proportion of office workers was 63.74%, and the proportion of patients with a monthly income of 6,000 yuan or more was 48.54%. Approximately 91.23% of the patients had medical insurance. Overall, 42.11% of the patients had a history of smoking, 61.99% had a history of drinking, and the majority of patients had a family history of chronic diseases, accounting for 51.46%.

**Table 1:** Basic characteristics of the surveyed population.

Basic characteristics	Experimental group (*N* = 171)	Control group (*N* = 171)	*P* value
Gender			
Man	154 (90.06%)	146 (85.38%)	0.188
Woman	17 (9.94%)	25 (14.62%)	
Age			
Average age (standard deviation) (years)	45.34 (16.60)	41.24 (16.60)	0.059
≥30	32 (18.1%)	51 (29.82%)	0.089
30–39	49 (28.65%)	39 (22.81%)	
40–49	28 (16.37%)	21 (12.28%)	
≥50	62 (36.26%)	60 (35.09%)	
Ethnic group			
Han ethnicity	160 (93.57%)	156 (91.23%)	0.414
Non-han ethnicity	11 (6.43%)	15 (8.77%)	
Marriage			
Married	132 (77.19%)	127 (74.27%)	0.528
Single	39 (22.81%)	44 (25.73%)	
Educational background			
Primary school and below	6 (3.51%)	4 (2.34%)	<0.001
Junior high school	25 (14.62%)	10 (5.85%)	
High school	41 (23.98%)	21 (12.28%)	
College degree or above	99 (57.89%)	136 (79.53%)	
Occupation			
Employed	109 (63.74%)	101 (59.06%)	0.081
Retired	42 (24.56%)	35 (20.47%)	
Unemployed	20 (11.70%)	35 (20.47%)	
Monthly income (RMB)			
≤3,000 YUAN	13 (7.60%)	34 (19.88%)	0.004
3,000–6,000 YUAN	75 (43.86%)	65 (38.01%)	
≥6,000 YUAN	83 (48.54%)	72 (42.11%)	
Medical insurance situation			
Yes	156 (91.23%)	158 (92.40%)	0.693
No	15 (8.77%)	13 (7.60%)	
History of smoking			
Yes	72 (42.11%)	52 (30.41%)	0.024
No	99 (57.89%)	119 (69.59%)	
History of drinking alcohol			
Yes	106 (61.99%)	82 (47.95%)	0.009
No	65 (38.01%)	89 (52.05%)	
Family history of chronic diseases			
Yes	88 (51.46%)	132 (77.19%)	<0.001
No	83 (48.54%)	39 (22.81%)	

(1) Single patients include those who are unmarried, divorced or widowed.

(2) Family history of chronic diseases includes: hypertension, diabetes, cerebral infarction, hyperuricemia, gout, and coronary atherosclerotic heart disease.

The control group consisted of 171 individuals, including those from the community and those who underwent health checks at the same hospital. The average age was 41.24 (16.60) years. The proportion of males was 85.38%, the Han population was 91.23%, the proportion of married individuals was 74.27, and 79.53% of the population had received higher education. The proportion of employed people and retirees was the greatest at 59.06 and 20.47%, respectively. Approximately 42.11% of the population had a monthly income of 6,000 yuan or more. The proportion of individuals with medical insurance was 92.4%. A history of smoking and drinking was reported for 30.41 and 47.95%, respectively. The majority of participants had a family history of chronic diseases, accounting for 77.19%.

We can also conclude from Table 1, that significant differences were present between the two groups in terms of educational background, monthly income (yuan), smoking history, drinking history, and family history of chronic diseases (*p* < 0.05, for all). In terms of occupation, marital status, ethnicity, and medical insurance, no significant differences were observed between the two groups of people (*p* > 0.05).

### 3.2 Reliability and validity of the scale

The Cronbach’s alpha coefficient of this scale was 0.895, indicating good reliability. Meanwhile, a structural validity analysis was conducted on the scale. The KMO value of this scale was 0.882 (>0.6), and the significance was *p* < 0.001. This study consisted of six dimensions, namely physical function, vitality, mental health, role limitation, social function, and pain. The principal component analysis method was used and, based on the rotated component matrix, the load of each question in only one dimension was considered >0.5, that is, each question was valid and passed the validity test. Therefore, the validity of this questionnaire is relatively good.

### 3.3 Measurement of HRQoL

**Figure 2:**
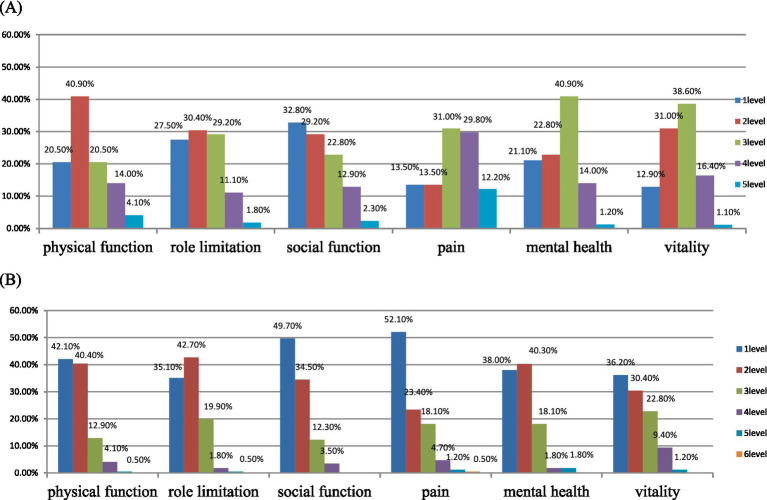
Distribution of different dimensions of the SF-6Dv2 scale. **(A)** Distribution of the affected population **(B)** Distribution of the control group population.

Among patients with hyperuricemia, 8.77% (*n* = 15) reported a state of complete health (code “111111”), and no one reported a state of worst health (code “555655”). The vitality dimension accounted for the highest proportion (87.1%) among the responses with no questions in the report, followed by the pain at 86.5%, the physical function at 79.5%, the mental health at 78.9%, and finally the role limitation and the social function dimensions at 72.5 and 67.2%, respectively (see Figure 2A). The average utility value of the patient group was 0.621 (0.270). In the subgroup classification based on demographic characteristics (Table 2), there were statistically significant differences in utility values for age, marital status, educational attainment, occupation, whether staying up late, triglyceride level, severity of hyperuricemia and gout, preference for seafood, and duration of hyperuricemia and gout (*p* < 0.05). At the same time, from the Table 2, we found that older the age, married status, lower the educational level, retired workers, hypertriglyceridemia, not staying up late, severity of hyperuricemia and gout, preference for seafood, duration of hyperuricemia and gout ≥1 year; The utility value of patients in this year was even lower, being lower than that of other people in the same group.

**Table 2:** Comparison of utility values between the two groups of people.

Factors	Experimental	*P* value	Control group	*P* value	*P* value *
	Average value (standard deviation)		Average value (standard deviation)		
Years					
≤30	0.653 (0.271)	*P* = 0.039	0.785 (0.184)	*P* = 0.607	P* = 0.018
30–39	0.697 (0.219)		0.806 (0.132)		P* = 0.005
40–49	0.594 (0.232)		0.840 (0.102)		P* < 0.001
≥50	0.556 (0.308)		0.803 (0.158)		P* < 0.001
Marriage					
Married	0.591 (0.280)	*P* = 0.013	0.817 (0.144)	*P* = 0.049	P* < 0.001
Single	0.720 (0.209)		0.763 (0.178)		P* = 0.255
Educational background					
High school and below	0.569 (0.281)	*P* = 0.032	0.776 (0.176)	*P* = 0.46	P* < 0.001
College degree or above	0.658 (0.257)		0.810 (0.149)		P* < 0.001
Occupation					
Employed	0.640 (0.258)	*P* = 0.016	0.846 (0.117)	*P* < 0.001	P* < 0.001
Retired	0.698 (0.257)		0.718 (0.187)		P* = 0.735
Unemployed	0.568 (0.282)		0.764 (0.175)		P* < 0.001
Monthly income (RMB)					
≤3,000	0.707 (0.237)	*P* = 0.167	0.763 (0.170)	*P* = 0.128	P* = 0.371
3,000–6,000	0.580 (0.289)		0.797 (0.152)		P* < 0.001
≥6,000	0.643 (0.254)		0.827 (0.148)		P* < 0.001
History of smoking					
Yes	0.600 (0.239)	*P* = 0.39	0.837 (0.146)	*P* = 0.055	P* < 0.001
No	0.635 (0.291)		0.788 (0.157)		P* < 0.001
History of drinking alcohol					
Yes	0.620 (0.270)	*P* = 0.972	0.844 (0.120)	*P* = 0.001	P* < 0.001
No	0.622 (0.273)		0.765 (0.174)		P* < 0.001
Stay up or no					
Yes	0.654 (0.259)	*P* = 0.036	0.795 (0.157)	*P* = 0.502	P* < 0.001
No	0.564 (0.281)		0.811 (0.154)		P* < 0.001
Have any chronic diseases or no					
Yes	0.574 (0.298)	*P* = 0.341			
No	0.671 (0.227)				
BMI					
BMI > 23.9	0.623 (0.271)	*P* = 0.841			
BMI ≤ 23.9	0.612 (0.271)				
Triglyceride levels					
Hypertriglyceridemia	0.479 (0.252)	*P* < 0.001			
Normal triglyceride level	0.771 (0.200)				
The severity of gout due to hyperuricemia					
Slight	0.898 (0.093)	*P* < 0.001			
Moderate	0.660 (0.161)				
Serious	0.403 (0.246)				
Like eating seafood or dislike					
Yes	0.469 (0.267)	*P* < 0.001			
No	0.751 (0.196)				
Duration of illness					
<1 year	0.737 (0.226)	*P* < 0.001			
≥1 year	0.513 (0.264)				

The calculation method for the P* value is: *t*-test for independent samples.

Being single includes: unmarried, divorced and widowed.

Chronic diseases of the body include: hypertension, diabetes, hyperlipidemia, coronary atherosclerotic heart disease, palpitations, kidney-related diseases, lumbar diseases, dizziness, and thyroid diseases.

The severity of gout due to hyperuricemia is classified based on the average uric acid level of patients in the past 2 months as stipulated in the “Multidisciplinary Expert Consensus on Diagnosis and Treatment of Hyperuricemia-Related Diseases in China (2023 Edition)” (29): Slight: 420–480 μmol/L; Moderate: 480–540 μmol/L; Serious: >540 μmol/L.

In the control group, a total of 7.01% (*n* = 12) of the respondents reported complete health status (code “111111”), and none reported the worst health status (code “555655”). Among the control group, the role limitation dimension had the highest proportion of the responses reporting any problem (64.9%), followed by the vitality dimension (63.8%), mental health dimension (62%), physical function dimension (57.9%), social function dimension (50.3%), and finally the pain dimension (47.9%) (see Figure 2B). In terms of the pain dimension, one respondent reported the most severe level, which was the sixth level of the pain dimension, “very severe pain”. The average utility value of the control group was 0.803 (0.155), which was better than that of the gout group with hyperuricemia. Compared with the patient group, the control group reported significantly lower levels in the six dimensions of physical function, role limitation, social function, pain, mental health, and vitality (*p* < 0.05).

As shown in Table 2, with regard to demographic characteristics, the utility values of the subgroups of the disease group were lower than those of the control group. Among the two groups, the subgroups with the greatest differences were those over 50 years of age (0.247), those who did not stay up late (0.247), followed by the 40–49-year-old group (0.246), those with a smoking history (0.237), and married individuals (0.226). Meanwhile, in the subgroup comparison between the disease group and the control group, significant differences were observed in the utility values of the two groups in terms of age, educational background, smoking history, drinking history, and staying up late (*p* < 0.05).

### 3.4 Factors related to health that influenced the quality of life

Table 3 reports the results of the multiple linear regression analysis to identify factors associated with quality of life. We found that patients whose marital status was single and who did not like seafood were positively correlated with a health utility value. That is, the value of health utility of patients who were single and did not like seafood was significantly higher, the *p*-values were 0.032 and < 0.001, respectively. However, the health utility values of patients with hypertriglyceridemia, hyperuricemia, and moderate and severe gout severity were negatively correlated. That is, among patients with hyperuricemia, compared with patients with moderate-to-severe gout and hyper- triglyceridemia, the health utility values of patients with hyperuricemia and with a gout severity of mild and with normal triglyceride levels were significantly higher. Therefore, patients who are single, dislike seafood, have mild hyperuricemia and gout, and have normal triglyceride levels have a higher health utility value compared to those who are married, like seafood, have moderate to severe hyperuricemia and gout, and suffer from hypertriglyceridemia, a finding consistent with the conclusion drawn above.

**Table 3:** Multiple linear regression analysis of the relationship between the utility values calculated using the SF-6Dv2 scale and demographic characteristics of participants, as well as the clinical variables.

Variables			Standardized coefficients	*t*	*P*-value
	*B*	SE	Beta		
(Constant)	0.838	0.089	0	9.454	0.000
Age (years) (Ref. ≤30 years)					
30–39	0.056	0.041	0.094	1.357	0.177
40–49	0.042	0.050	0.058	0.847	0.398
≥50	−0.007	0.047	−0.012	−0.149	0.881
Woman (Ref. Man)	−0.081	0.048	−0.090	−1.692	0.093
Non-han ethnicity (Ref. Han ethnicity)	−0.012	0.055	−0.011	−0.214	0.831
Single (Ref. Married)	0.080	0.037	0.125	2.167	0.032
Medical insurance (Ref. Yes)	0.032	0.049	0.034	0.658	0.511
No stay up (Ref. Stay up)	−0.047	0.040	−0.084	−1.159	0.248
Hypertriglyceridemia (Ref. Normol trig lyceride level)	−0.128	0.032	−0.238	−4.020	0.000
Dislike eating sea food (Ref. Like eating seafood)	0.123	0.030	0.229	4.163	0.000
College degree or above (Ref. High school and below)	−0.021	0.036	−0.039	−0.587	0.588
No smoking (Ref. smoking)	0.039	0.029	0.072	1.371	0.172
No drinking (Ref. drinking)	−0.023	0.029	−0.042	−0.794	0.428
BMI > 23.9 (Ref. BMI ≤ 23.9)	0.039	0.039	0.058	1.000	0.319
Occupation (Ref. employed)					
Unemployed	−0.104	0.059	−0.124	−1.745	0.083
Retired	0.018	0.042	0.029	0.436	0.663
Time of illness ≥ 1 year (Ref. time of illness < 1 year)	0.026	0.033	0.049	0.795	0.428
The severity of gout due to hyperuricemia slight (Ref. Slight)					
Moderate	−0.132	0.043	−0.236	−3.095	0.002
Severe	−0.345	0.051	−0.623	−6.756	0.000
Monthly income (RMB) (Ref. ≤ 3,000)					
3,000–6,000	−0.116	0.068	−0.215	−1.721	0.087
≥6,000	−0.082	0.072	−0.152	−1.13	0.256
Adjusted *R*^2^	0.629				

## 4 Discussion

In this study, we used the SF-6Dv2 scale to assess the HRQoL of the patient population with gout and hyperuricemia in the Tianjin area of China for the first time and compared it with that of the general population. In addition, this study also explored the correlation between HRQoL and population demographics and clinical factors. According to a review of the relevant literature, this is the first study to use the SF-6Dv2 scale to assess the HRQoL of gout patients with hyperuricemia in the Chinese population and compare it with that of the general population. The results of this study will be used to supplement the understanding of the HRQoL data of the gout population with hyperuricemia, providing empirical data for the pharmacoeconomic evaluation in the Tianjin area of China.

In this study, no ceiling or floor effect was found, which is consistent with the reports in the relevant literature (22, 23). The average utility value of the patient group was 0.621 (0.270), which was significantly lower than that of the control group at 0.803 (0.155), *p* < 0.001. Through multiple linear regression analysis, a linear correlation was found between single marital status, presence of hypertriglyceridemia, preference for eat seafood, and the severity of gout due to hyperuricemia of patients and the utility value of the SF-6Dv2 scale. Gout is a metabolic disorder that can be controlled but not cured. Therefore, it has a negative impact on the quality of life of the patient in the long-term. If not well controlled, the disease will continue to develop, thus affecting quality of life from multiple dimensions.

By comparing the utility values of the subgroups of the disease group, the utility value of patients aged ≥50 years was lower than that of patients in other age groups. The potential reasons were as follows. Older patients often have other chronic diseases, and their physical condition and energy vitality are poorer than those of younger and middle-aged patients. The utility value of patients with married marital status was lower than that of patients with single status. The possible reasons are that patients with married status live with more family members and thus, they need to take into account the tastes of many people in terms of diet. In particular, the health behavior and lifestyle of the spouse can have an impact on patients. At the same time, the numerous household chores may lead married patients to neglect their own health problems. The utility value of patients with high school and lower education was less than that of people with a higher level of education. This might be due to the fact that self-discipline in life was not as rigorous as that of other members of the subgroup and may have poorer control over diet and exercise, thus resulting in less effective disease control. Retired people had relatively less regular daily routines compared with those of other groups, which can also affect the disease progression and, in turn, their quality of life. In this study, the utility value of the unemployed was higher than that of the employed. Employed individuals usually have higher work pressure and longer high-intensity working hours, which may lead to an increase in the psychological stress of the patients and thereby affect the utility value. Unemployed individuals may experience better sleep, more regular and healthy exercise, and less psychological stress, so the utility value is higher. In terms of monthly income, patients with a monthly income ranging from 3,000 to 6,000 yuan have an income level below average. They bear relatively greater economic pressure in life and thus their quality of life is also lower than that of people with higher incomes. Nonetheless, patients with a monthly income of less than 3,000 yuan have a relatively better quality of life. Potential reasons include reduced mental stress from work compared with that of individuals with higher incomes, despite their economic level is not good. The utility value of patients with a history of smoking and drinking is lower than that of patients with no history of smoking and drinking. Because these patients present a greater lifestyle risks, an increased metabolic burden on the liver and kidneys, raises the risk of other chronic diseases (such as lung and heart diseases) in the long term, albeit smoking and drinking can relieve stress, and also increase the cost of living. The utility value of the scale of patients with hypertriglyceridemia was lower than that of patients with normal triglycerides, which is consistent with reports in the literature (26, 27). Uric acid levels are correlated with dyslipidemia, and even in patients with gout, it can affect lipid metabolism levels. In this study, the utility value of patients with BMI ≤ 23.9 was better than that of patients with BMI > 23.9, which is inconsi- stent with the existing literature (28). Obesity is also a cause of hyperuricemia and gout. Many factors affect the utility value defined by the patient, including diet, obesity index, blood lipid level, and exercise status. In this study, many patients had the habit of staying up late. Therefore, whether or not they stayed up late was taken as a clinical influencing factor in this study. However, the results showed that the utility value of patients who stayed up late was better than that of those who did not stay up late. Staying up late may thus also affect the patient’s mood and the progression of the disease. Meanwhile, no relevant literature is currently available describing staying up late as a clinical influencing factor for hyperuricemia and gout. Therefore, further clinical research is still needed.

In this study, evaluating the utility values of the SF-6Dv2 scale for patients with different demographic and clinical characteristics and conducting subgroup analyses helped better understand the differences in utility values among different subgroups and also provided a parameter for the utility values of the different health status scores of patients with gout and hyperuricemia. Utility values vary between different countries and different populations. Using utility values calculated by other countries may lead to deviations in the assessment of future cost-effectiveness analysis of health economics for patients with hyperuricemia and gout in China. However, the calculation of the utility values of the SF-6Dv2 scale used in this study was based on preference weights of the Chinese population (25). Thus, we propose this scale as suitable for use among the Chinese population. This study supports the use of the SF-6Dv2 scale to obtain a more accurate assessment of the health outcomes of patients with hyperuricemia and gout in the future. Furthermore, the scale scores can provide reliable first-hand data to researchers, and thus better informed decision-making by the health department in the allocation of medical and health resources.

This study has some shortcomings that should be acknowledged. First, this study was conducted in a specific city in China and no research was carried out across multiple cities. Additional multicenter studies are needed to investigate the applicability of the scale to measure the quality of life of gout patients with hyperuricemia at a national level. Second, this study was based on population from a large tertiary hospital. No patients with gout and hyperuricemia were enrolled from the community. This might be due to milder clinical conditions of the patient or to the inconvenience of seeking medical treatment. Finally, other potential factors not addressed in this study may affect the HRQoL of patients. Due to the limitations of research time and subjects, the collection of influencing factors, such as medication use, diet, living habits, and preference for exercising, was not exhaustive. These factors were not included in this study and need to be confirmed by further research.

## 5 Conclusion

This study used the SF-6Dv2 scale for the first time to assess the quality of life related to gout patients with hyperuricemia in Tianjin, China, and compared with the quality of life of the general population. The study revealed the current situation of the HRQoL of patients and their influencing factors, providing a basis to more accurately formulate a path of clinical treatment for hyperuricemia and gout in the future in China.

## Data Availability

The original contributions presented in the study are included in the article/Supplementary material, further inquiries can be directed to the corresponding authors.
